# Sevoflurane Exacerbates Cognitive Impairment Induced by A*β*_1–40_ in Rats through Initiating Neurotoxicity, Neuroinflammation, and Neuronal Apoptosis in Rat Hippocampus

**DOI:** 10.1155/2018/3802324

**Published:** 2018-10-09

**Authors:** Yue Tian, Ke-yan Chen, Li-dan Liu, Yun-xia Dong, Ping Zhao, Shan-bin Guo

**Affiliations:** ^1^Department of Anesthesiology, Shengjing Hospital of China Medical University, No. 36, Sanhao Street, Herping District, Shenyang, Liaoning 110004, China; ^2^Department of Laboratory Animal Science, China Medical University, No. 77, Puhe Road, Shenyang North New Area, Shenyang, Liaoning 110122, China; ^3^Department of Pharmacy, Shengjing Hospital of China Medical University, No. 36, Sanhao Street, Herping District, Shenyang, Liaoning 110004, China

## Abstract

**Objective:**

This study was aimed at investigating whether sevoflurane inhalation induced cognitive impairment in rats with a possible mechanism involved in the event.

**Methods:**

Thirty-two rats were randomly divided into four groups of normal saline (NS) + O_2_, NS + sevoflurane (sevo), amyloid-*β* peptide (A*β*) + O_2_, and A*β* + sevo. The rats in the four groups received bilateral intrahippocampus injections of NS or A*β*. The treated hippocampus was harvested after inhaling 30% O_2_ or 2.5% sevoflurane. Evaluation of cognitive function was performed by Morris water maze (MWZ) and an A*β*_1–42_ level was determined by ELISA. Protein and mRNA expressions were executed by immunohistochemical (IHC) staining, Western blotting, and qRT-PCR.

**Results:**

Compared with the NS-treated group, sevoflurane only caused cognitive impairment and increased the level of A*β*_1–42_ of the brain in the A*β*-treated group. Sevoflurane inhalation but not O_2_ significantly increased glial fibrillary acidic protein (GFAP) and ionized calcium-binding adaptor molecule (IBA)1 expression in A*β*-treated hippocampus of rats. Expression levels for Bcl-xL, caspase-9, receptor for advanced glycation end products (RAGE) and brain-derived neurotrophic factor (BDNF) were significantly different in quantification of band intensity between the rats that inhaled O_2_ and sevoflurane in A*β*-treated groups (all *P* < 0.05). Interleukin- (IL-) 1*β*, nuclear factor-*κ*B (NF-*κ*B), and inducible nitric oxide synthase (iNOS) mRNA expression increased after the rats inhaled sevoflurane in the A*β*-treated group (both *P* < 0.01). There were no significant differences in the change of GFAP, IBA1, Bcl-xL, caspase-9, RAGE, BDNF, IL-1*β*, NF-*κ*B, and iNOS in the NS + O_2_ and NS + sevo group (all *P* > 0.05).

**Conclusion:**

Sevoflurane exacerbates cognitive impairment induced by A*β*_1–40_ in rats through initiating neurotoxicity, neuroinflammation, and neuronal apoptosis in rat hippocampus.

## 1. Introduction

The hippocampus, a brain area critical for learning and memory, is especially vulnerable to damage at early stages of Alzheimer's disease (AD) [1]. Some evidence support that amyloid-*β* (A*β*) peptide is a key trigger of AD pathogenesis [2]. It has been found that the A*β*-associated increases in bone morphogenetic protein 6 (BMP6) expressions were accompanied by reduced markers of neurogenesis in the hippocampus of patients with AD [3]. A*β* is obtained by hydrolysis of its precursor protein APP, and only aggregated A*β* is neurotoxic, leading to synaptic dysfunction and impaired memory. Injection of aggregated A*β* polypeptide fragments into the brain of rats can mimic the development of senile plaque in the brain of AD patients, cause damage to the nervous system, and lead to cognitive impairment in experimental animals.

The incidence of AD was approximately 13% in people over 65 years of age, and about 66 million patients over the age of 65 require surgery per year worldwide. In other words, nearly 8.5 million AD patients require anesthesia each year [4]. In recent years, the potential neurotoxicity of inhaled anesthetics has attracted the attention of many researchers. Older people are more likely to have higher CNS complications after anesthesia surgery. Neurotoxicity of inhaled anesthetics is very similar to that of AD-related pathologies. Studies have shown that the use of halogen anesthetics, including sevoflurane, increases the oligomerization/deposition of A*β* and the phosphorylation of tau protein [5]. However, the existing epidemiological studies cannot conclude that the toxicity of anesthetics to the central nervous system is an independent risk factor for AD. A meta-analysis of 15 case-control studies showed no association between anesthetic exposure and AD [6]. In recent years, the impact of inhaled anesthetics on the elderly population has drawn attention [7] and also needed further data to explore.

Sevoflurane is an inhaled volatile anesthetic agent that is widely used in pediatric practice [8]. Studies have shown that the sevoflurane induces neuroinflammation by upregulating the expression of inflammatory cytokines such as tumor necrosis factor-*α* (TNF-*α*) and interleukin- (IL-) 1*β* and resulted in the increase of A*β* expression, hyperphosphorylation of tau, neuronal apoptosis, and subsequent cognitive impairment [9–11]. The effects of sevoflurane on cognitive ability were reported in aged rats [11, 12]. In the elderly population, there were many AD patients. Therefore, it is necessary to explore the effect of sevoflurane on cognitive function and pathological changes of the brain in AD patients and in this study we used the A*β*-treated rats to imitate the AD patients.

Therefore, the purpose of this study was to investigate whether sevoflurane inhalation impaired cognitive function with a possible mechanism(s) in AD model rats established by A*β* injection. Our findings not only provide information regarding the effects of sevoflurane on the A*β*-injected hippocampus but also suggest that cognitive impairment is due to multiple mechanisms leading to hippocampal dysfunction.

## 2. Materials and Methods

### 2.1. Animals

Male Sprague-Dawley (SD) rats weighting 230–280 g were purchased from the Experimental Centre of Animals at China Medical University. The animals at 12 weeks of age were housed in an environmentally controlled animal facility of the center for the duration of the experiments. Thirty-two rats were randomly divided into four groups including normal saline (NS) + O_2_, NS + sevoflurane (sevo), A*β* + O_2_, and A*β* + sevo. We took 8 rats from each group to perform the experiment. Another four rats were taken from each of the NS + sevo and A*β* + sevo groups to test the vital signs of sevoflurane inhalation and then sacrificed after the test. All procedures have been reviewed and approved by the Research Review Committee of China Medical University.

### 2.2. Bilateral Intrahippocampus Injection and Sevoflurane Inhalation

Oligomeric A*β*_1–40_ solution (Sigma, USA) was prepared for rats receiving bilateral intrahippocampus injections at a concentration of 5 *μ*g/*μ*l. The solution was incubated in an incubator at 37°C for one week and stored at 4°C until use. The sites of injection were selected at the hippocampal CA1 region in the right and left anterior positions (3.0 mm posterior to the bregma, 2.2 mm lateral to the midline, and 3.5 mm beneath the surface of the skull). Two stainless-steel syringes were inserted through a guide cannula to produce 0.5 mm below the tips of the latter. Then, aliquots of 2 *μ*l of NS or A*β*_1–40_ were gently injected into both sides of the CA1 region within 5 min, according to our protocol. The needles were left in place for an additional 10 min to allow for diffusion of the injected solution. Before the injection of A*β*_1–40_ or NS, the rats were anesthetized by intraperitoneal injection of 10% chloral hydrate (250 mg/kg).

The rats in each group were exposed to 2.5% sevoflurane or 30% O_2_ for 4 hours after 14 days of injection of NS or A*β*. The method of sevoflurane inhalation was described as follows: the rats inhaled sevoflurane using a self-made anesthesia chamber (50 cm × 30 cm × 30 cm). The anesthesia chamber was kept at 37°C warm water bath and had two ventilating holes inside: the superior one connected to an anesthesia pump and the inferior one connected to a gaseous monitor. The rats in the NS + sevo group and A*β* + sevo group were anesthetized with 5% sevoflurane in the anesthesia chamber firstly. After 2 minutes, the rats were unconscious, and then the rats were treated by 2.5% sevoflurane and 30% O_2_ for 4 h (flow rate = 1.5 l/min). Rats in the NS + O_2_ group and A*β* + O_2_ group were given only 30% oxygen inhalation for 4 hours in the anesthetic chamber.

### 2.3. Observation of Vital Signs of Sevo Inhalation

Some of the NS and A*β*-treated rats (*n* = 4) were lightly anesthetized with sevoflurane by an anesthesia machine (MATRX, US) when inhaling 2.5% sevoflurane for 4 hours. The rats were surgically treated with catheterization inserted into a carotid artery, and the other end was connected to a pressure transducer (ADInstruments, New Zealand). Hemodynamic parameters (mean arterial pressure (MAP), heart beats, pH, PaCO_2_, PaO_2_, and HCO_3_^−^) of the rats were continuously monitored for 4 hours using a blood gas analyzer (GE, US) when inhaling 2.5% sevoflurane. These rats were used to explore the impacts of sevoflurane on the vital signs of the rat model, and they were sacrificed after the test.

**Figure 1:**
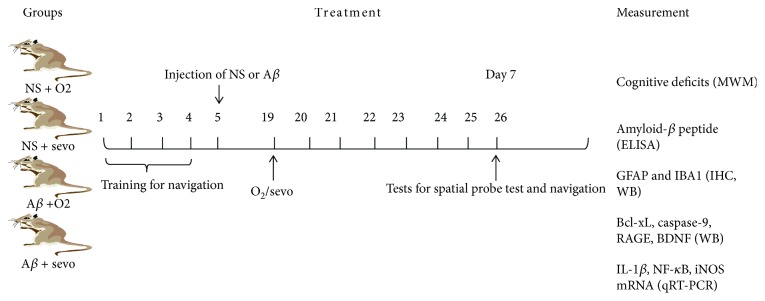
Schematic representation of study designs. Animal model of Alzheimer disease was established with injection of NS or A*β* on day 7. Those animals started to inhale 30% O_2_ or 2.5% sevoflurane after 14 days of the above injection.

A concentration of sevoflurane was selected for the animals according to the previous studies (13–15). The rest of the rats (32 rats) were followed according to our protocol and started to inhale O_2_ or sevoflurane for 4 hours using a self-made anesthesia chamber on day 14 after injections of NS or A*β* (Figure 1).

### 2.4. Morris Water Maze Experiment

To detect the difference in the learning and memory ability of rats in each group before the AD model being established, Morris water maze (MWZ) test was performed. The main component of the water maze was a round pool (diameter: 160 cm, height: 50 cm, and depth: 35 cm), and it was divided into four equal quadrants (I, II, III, and IV) according to 4 equidistant points on the wall of the pool. The platform was placed under 2 cm of the surface of the water in the center of quadrant IV, and the diameter of the platform is 15 cm. The methods of navigation test were described as follows: the rats were placed in pools from 4 different quadrants and faced the pool wall, and then all rats were allowed to swim freely until the platform which was hidden beneath the water was found. The swimming speed and escape latency (the time from which the rats entered the water to which the rats climbed to the platform) were recorded by the Any-Maze video-tracking system (Yishu Info, China). If the rat did not find the platform within 90 seconds, they will be guided to the platform to rest for 30 seconds and the escape latency was recorded as 90 s. The training was performed 4 days and 4 times per day. 1 day after the navigation test was finished, the rats in the four groups received bilateral intrahippocampus injections of NS or A*β*. And then, the rats in each group were exposed to 2.5% sevoflurane or 30% O_2_ for 4 hours after 14 days of injection of NS or A*β*. At 7 days after exposure, all rats were subjected to the second MWZ. The platform was removed, and spatial probe test was performed on all the rats and the time spent in exploration of the original platform was recorded. Then, the platform was moved to the opposite side of the original quadrant (quadrant II). All rats received 4 times navigation test for the new platform. Before the test, the rats were placed on a new platform for 30 s. Then, the test was performed according to the above method, and the speed of swimming and escape latency were recorded. Take the average of 4 results as the experimental result for each animal. Performances of navigation on the fourth day were recorded as the pretreatment scores.

### 2.5. Determination of Protein Products

Experimental animals were sacrificed and the hippocampus was surgically removed. Samples of hippocampal regions from the grouped animals (NS + O_2_, NS + sevo, A*β* + O_2_, and A*β* + sevo) were harvested and immediately dissolved in ice normal saline (25 ml/g). Hippocampus tissues were ground using a homogenizer at approximately 4°C. The supernatant from each group was obtained with centrifugation (2500 r/min × 15 min) at 4°C and placed in a clean plastic screw-cap vial for ELISA. The protein concentration of the supernatant was determined by NanoDrop 2000 spectrophotometer (Thermo Scientific, US).

A*β*_1–42_ peptide in the hippocampus tissue of the grouped animals was identified using an antibody against the murine A*β* (dilution, 1 : 200) from Santa Cruz (CA, US). The expression level of protein in the supernatant from the tissue was measured using ELISA according to the manufacturer's directions (Shanghai Xi Mei Chemical Co. Ltd., China). Briefly, 100 *μ*l of the substrate solution was added to 100 *μ*l of the sample in microtiter plates in duplicate tests and then incubated for 30 min at 37°C. The reaction was stopped by adding 50 *μ*l of 4 M sulfuric acid, and the OD values were read in a microtiter autoreader at 450 nm.

### 2.6. Immunohistochemical Staining

We took 100 mg hippocampus tissue to perform immunohistochemical staining. Some of the tissues were fixed with formaldehyde (10%) for 24 hours and then embedded in paraffin before cutting into 5 *μ*m sections. For IHC staining, hippocampus tissues were infiltrated in PBS containing 4% (*v*/*v*) paraformaldehyde for 30 min and then dehydrated and embedded using paraffin. Sections (5 *μ*m) of the specimens were made, and they were taken care to prevent overstretching. The sections were incubated with rabbit monoclonal anti-glial fibrillary acidic protein (GFAP) (dilution, 1 :1 000) or ionized calcium-binding adaptor molecule 1 (IBA1) (dilution, 1 : 500) antibody from Santa Cruz (CA, US) as the primary antibody for at least 18 hours at 4°C. A horseradish peroxidase-conjugated goat anti-rabbit IgG antibody (dilution, 1 : 500) was incubated as the secondary antibody for 1 hour at room temperature after the sections were washed with PBS. The sections were photographed using light microscopy (Olympus, Japan) with amplification at 400x.

### 2.7. Electrophoresis and Quantification of Proteins

Specimens of the **hippocampal** region were taken from the grouped animals. Aliquots (10 *μ*g/well) of **hippocampal** lysate were size-fractionated on a 4–20% SDS-PAGE gel and transferred to a nitrocellulose membrane and blocked with 5% nonfat dried milk in TBS. The membrane was individually incubated with anti-GPAP (dilution, 1 : 500), anti-IBA1 (dilution, 1 : 5 00), anti-Bcl-xL (dilution, 1 : 1000), anti-caspase-9 (dilution, 1 : 2000), anti-receptor for advanced glycation end products (RAGE) (dilution, 1 : 1000) or anti-brain-derived neurotrophic factor (BDNF) (dilution, 1 : 1000) (Santa Cruz, CA), and *β*-actin (dilution, 1 : 500) (BioVision, US) as the primary antibodies at 4°C overnight and then incubated with horseradish peroxidase-conjugated secondary antibody (dilution, 1 : 2000) after washing with TBS. Proteins were quantified using gel image analysis system and relative band intensity was calculated as % of the intensity of the *β*-actin protein band.

### 2.8. Measurement of IL-1*β*, Nuclear Factor-*κ*B (NF-*κ*B), and Inducible Nitric Oxide Synthase (iNOS) mRNA

Specimens of the **hippocampal** region were taken from the grouped animals on day 7 following the last exposure and homogenized using a rapidly oscillating masher. IL-1*β*, NF-*κ*B, and iNOS mRNA expression levels in the region were assessed by using SYBR Premix Ex TaqTM (Takara, Japan). The following forward primers for the Il-1*β*, NF-*κ*B, iNOS, and *β*-action mRNA were used a sequence in 5′-GCTGTGGCAGCTACCTATGTCTTG-3′, 5′-ACTGCCGGGATGGCTTCTAT-3′, 5′-TCCACCTCCTTCCCTGAACTGG-3′ 5′-CTTAGTTGCGTTACACCCTTTCTTG-3′. Reverse primers for the IL-1*β*, NF-*κ*B, iNOS, and *β*-action mRNA were 5′-AGGTCGTCATCATCCCACGAG-3′, 5′-CTGGATGCGCTGGCTAATGG-3′, 5′-TGATGACGGTGATGAAGAATAT-3′, 5′-CTGTCACCTTCACCGTTCCAGTTT-3′. Briefly, total RNA in the tissues was extracted using a tissue homogenizer in Qiagen lysis buffer (Qiagen, UK), and purification of RNA was performed with Qiagen RNeasy minicolumns (Qiagen, UK) following the manufacturer's protocol. RNA was quantified using the NanoDrop ND-1000 spectrophotometer (Thermo Fisher, USA) and amplified and biotin-labeled with NuGEN's Ovation System, according to the manufacturer's instructions (Roche, Switzerland). The yield of the total RNA per replicate varied from 0.6 *μ*g to 2.0 *μ*g. 50 ng of the RNA was added in a SYBR qPCR master mix for real-time RT-PCR. The units from target gene expression in the grouped samples were normalized by the *β*-actin value. Contents of IL-1*β*, NF-*κ*B, and iNOS mRNA were expressed as a fold change as compared to the expression level of *β*-actin mRNA.

### 2.9. Statistical Analysis

Values were expressed as mean ± standard deviation (SD) on the results. Statistical analysis was performed using Statistical Package for the Social Science (SPSS) version 21.0 (SPSS, IL). Student *t*-test was used to compare measurements of two groups. A *P* value of <0.05 was considered statistically significant.

## 3. Results

### 3.1. Vital Sign Measurement

**Figure 2:**
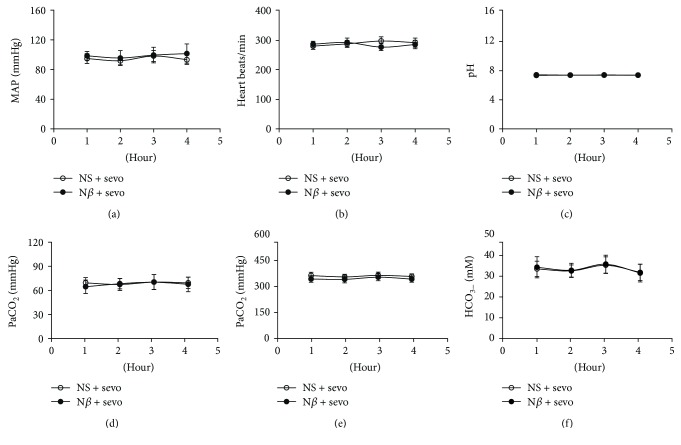
Effects of sevoflurane on animal health. To verify whether inhalation anesthesia with sevoflurane was toxic to animals, the NS- and A*β*-treated rats inhaled 2.5% sevoflurane for 1–4 hours, and then MAP (a), heart beats (b), pH (c), PaCO_2_ (d), PaO_2_ (e), and HCO_3_^−^ (f) were continuously monitored in the animals. There were no statistical significance in the above-stated parameters among 4 different time points in the NS + sevo group or A*β* + sevo group (all *P* > 0.05 and *n* = 4 in each group).

To check whether inhalation of sevoflurane was toxic to animals, 4 rats in the NS + sevo group and A*β +* sevo group were monitored with the inhaled gas in the preexperimental study. The data were collected during the length of 1–4 hours of time, and the results are shown in Figure 2. 2.5% sevoflurane inhalation alone did not show significant differences in measurements of MAP (Figure 2(a)), heart beats (Figure 2(b)), pH (Figure 2(c)), PaCO_2_ (Figure 2(d)), PaO_2_ (Figure 2(e)), and HCO_3_^**−**^ (Figure 2(f)) among 4 different time points (1, 2, 3, and 4 h) for the NS + sevo group (all *P* > 0.05). The similar results were obtained among 4 different time points (1, 2, 3, and 4 h) in the A*β* + sevo group. These results indicated that 2.5% sevoflurane inhalation for 4 hours had no effect on the vital signs of NS and A*β*-treated rats and no hypoxia or carbon dioxide accumulation. The concentration and the length of time for sevoflurane inhalation were feasible and appropriate in the animals, which did not find toxic effects in vital sign monitoring.

### 3.2. Effects of Sevoflurane on Cognitive Ability

**Figure 3:**
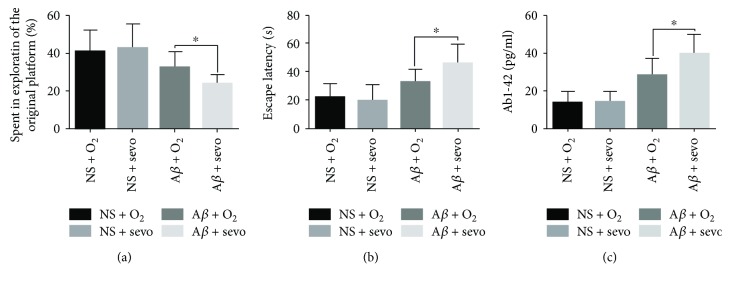
Sevoflurane influenced cognitive function and A*β*_1–42_ level in rats. Rats received bilateral intrahippocampus injections of either NS or A*β*_1–40_ after training. The spatial probe test was examined in the time course after inhalation of 30% O_2_ or 2.5% sevoflurane, and the time spent in exploration of the original platform was recorded (a). The results were expressed as a ratio (%) as compared to the NS + O_2_ or A*β* + O_2_ group on day 7. Navigation test was performed after inhalation of 30% O_2_ or 2.5% sevoflurane on day 7, and the escape latency was recorded (b). The results were expressed as seconds as compared to the NS + O_2_ or A*β* + O_2_ group at the given times. After the rats inhaled 30% O_2_ or 2.5% sevoflurane in the presence and absence of A*β*, hippocampus tissues of the treated rats were harvested. And then, the level of A*β*_1–42_ in the supernatant from the tissue was determined by ELISA (c). ^∗^*P* < 0.05 vs. A*β* + O_2_ (*n* = 8).

Spatial probe test and navigation test were performed to determine the effect of sevoflurane on the cognitive ability since the hippocampal formation should be the first brain region to exhibit neurodegeneration in AD, and determination of AD-related alterations in hippocampal function should be central of the AD diagnosis. For the spatial probe test and navigation test, the time spent in exploration of the original platform and the escape latency were recorded respectively on day 7 in four groups (Figures 3(a) and 3(b)). The results showed that there were no differences in the time spent in exploration of the original platform and escape latency between the NS + O_2_ and NS + sevo group on day 7 (*P* > 0.05, Figures 3(a) and 3(b)). In contrast to NS-treated groups, there were significant differences in the time spent in exploration of the original platform and escape latency on day 7 between the A*β* + O_2_ and A*β* + sevo group (*P* < 0.05, Figures 3(a) and 3(b)). The results indicated that the A*β*-treated rats with sevoflurane inhalation displayed worse performance of cognitive ability. No significant differences in the performances of navigation on the fourth day were recorded as the pretreatment scores in the 4 groups.

### 3.3. A*β*_1–42_ Level in Hippocampus

For understanding the effect of sevoflurane on AD, experimental rats received bilateral intrahippocampus injections of NS or A*β*_1–40_ and then inhaled 30% O_2_ or 2.5% sevoflurane, respectively. The average values (pg/ml) for the A*β*_1–42_ protein product in hippocampus tissues harvested on day 7 were shown in Figure 3(c). The A*β*_1–42_ level in the hippocampus of the A*β*-treated rats with sevoflurane inhalation was obviously higher than that in the O_2_ exposure group (*P* < 0.05, Figure 3(c)).

### 3.4. Expression of GFAP and IBA1 in Hippocampus

**Figure 4:**
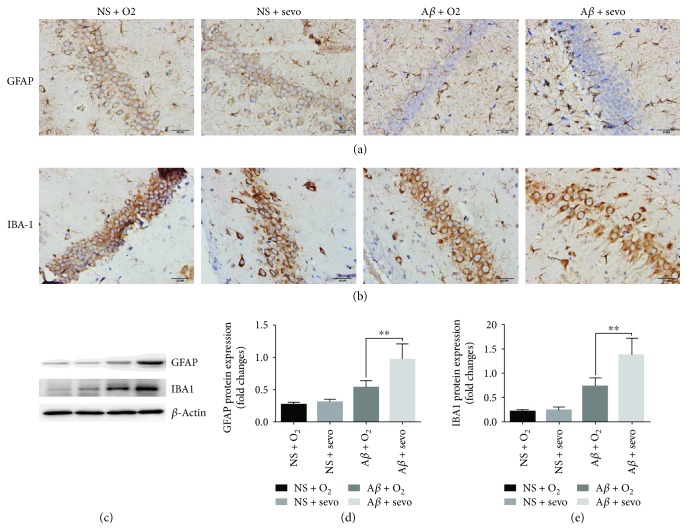
Sevoflurane inhalation increased protein expression of GFAP and IBA-1 in the hippocampus determined by immunochemistry and Western blot. Immunochemistry was used to determine the GFAP (a) and IBA1 (b) expression levels in the NS- or A*β*-treated hippocampus of rats after inhaling 30% O_2_ and 2.5% sevoflurane at the indicated times. Western blot was also used to determine the expression of GFAP and IBA1 in the four groups (c). The results in (d) and (e) were calculated as % of the intensity of the *β*-actin protein band and expressed as a fold change in reference to their controls. ^∗∗^*P* < 0.01 vs. A*β* + O_2_ (*n* = 8).

Protein expression in rat hippocampus was examined using an IHC method in the presence of 30% O_2_ and 2.5% sevoflurane. The results are shown in Figure 4. Positive responses of GFAP and IBA1 proteins to their antibodies were observed in the tissues stained in dark brown color in the images (Figures 4(a) and 4(b)). Astrocytes and microglia with the expression of GFAP and IBA1 proteins are distributed in all CA1 layers of the hippocampus. Then, we performed the Western blotting to determine the expression level of GFAP and IBA1. There were significantly higher expressions of GFAP and IBA1 in the A*β* + sevo groups compared to the A*β* + _*O*2_ groups (*P* < 0.01, Figures 4(c)–4(e)). No significant differences were found between the NS + _O2_ and NS + sevo groups on day 7 (*P* > 0.05). These indicated that sevoflurane inhalation obviously increased GFAP and IBA1 protein expression in the A*β*-treated hippocampus of rats at the indicated times.

### 3.5. Expression of Bcl-xL, Caspase-9, RAGE, and BDNF in Hippocampus

**Figure 5:**
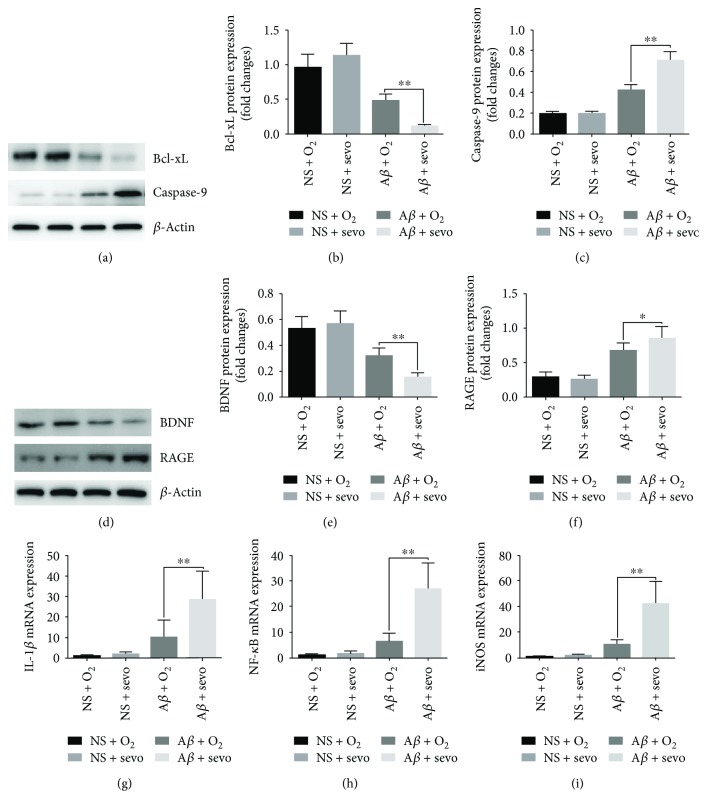
Sevoflurane inhalation influenced protein expression of Bcl-xL, caspase-9, BDNF, RAGE, and mRNA level of IL-1*β*, NF-*κ*B, and iNOS in the hippocampus. Caspase-9 (a, b), Bcl-xL (a, c), BDNF (d, e), and RAGE (d, f) protein levels in the NS- or A*β*-treated hippocampus of rats were determined using Western blot after inhaling 30% O_2_ and 2.5% sevoflurane at the indicated times. Relative band intensity of each protein expression was calculated as % of the intensity of the *β*-actin protein band and expressed as a fold change in reference to their controls. IL-1*β* (g), NF-*κ*B (h), and iNOS (i) mRNA levels in the NS- and A*β*-treated hippocampus were determined after inhaling 30% O_2_ or 2.5% sevoflurane on day 7. The results were expressed as a fold change in reference to mRNA levels of their own controls. ^∗^*P* < 0.05 vs. A *β* + O_2_ (*n* = 8); ^∗∗^*P* < 0.01 vs. A *β* + O_2_ (*n* = 8).

Bcl-xL, caspase-9, RAGE, and BDNF protein expressions in the hippocampus of rats were examined using Western blotting analysis. The results are shown in Figure 5. The intensities of caspase-9 (Figures 5(a) and 5(c)) and RAGE (Figures 5(d) and 5(f)) proteins in the A*β*-injected hippocampus of rats that received sevoflurane were significantly higher than those of the rats exposed to O_2_ on day 7 (all *P* < 0.05). However, the intensities for Bcl-xL (Figures 5(a) and 5(b)) and BDNF (Figures 5(d) and 5(e)) proteins in the hippocampus of the A*β*-injected rats exposed to sevoflurane were obviously lower than those of the rats with O_2_ inhalation on day 7(all *P* < 0.05). No significant differences in the expression of Bcl-xL, caspase-9, RAGE, and BDNF between the NS + O_2_ and NS + sevo groups on day 7 (all *P* > 0.05).

### 3.6. IL-1*β*, NF-*κ*B, and iNOS mRNA Expression in Hippocampus

IL-1*β*, NF-*κ*B, and iNOS mRNA expressions in NS- and A*β*-injected hippocampus of rats were determined on day 7. IL-1*β*, NF-*κ*B, and iNOS mRNA levels were calculated as a fold change in reference with the expression level of *β*-actin mRNA. The results of IL-1*β*, NF-*κ*B, and iNOS expression on day 7 were shown in Figure 5. Significant differences were found in the three gene expressions between A*β* + O_2_ and A*β* + sevo groups (all *P* < 0.01, Figures 5(g)–5(i)).

## 4. Discussion

Some hypotheses attribute the delayed neurocognitive deficits produced by sevoflurane to direct neurotoxic effects [6–17]. Potential risks were examined in rats that received 30% O_2_ or 2.5% sevoflurane for 4 hours after the hippocampus of rats was bilaterally injected with NS or A*β*, and the 2.5% sevoflurane only has neurotoxic effects to rats injected with A*β* in the hippocampus. Our results revealed that vital signs including measurements of MAP, heart beats, pH, PaCO_2_, PaO_2_, and HCO_3_^**−**^ were not obviously changed at 4 different time points (1, 2, 3, and 4 h) for the NS + sevo group or A*β* + sevo group, suggesting that intervention of sevoflurane was not toxic to these rats at duration and concentration of the inhaled anesthetic in the animal study. These clinical observations not only displayed the quality initiative in the clinical practice of anesthesiology but also set up fundamental steps in the experimental design for further studies.

Inhalation anesthesia with sevoflurane but not O_2_ in A*β*-treated rats resulted in obvious increases in the length of time for escape latency, indicating that sevoflurane inhalation was able to induce neurocognitive dysfunction in the rats as assessed by the MWM. This result was consistent with other research on the escape latency from the hidden platform [18]. Anesthetic drugs may cause widespread neurodegeneration in the developing brain and persistent learning deficits linked to anesthesia-induced neurotoxicity [19, 20]. Although inhalation anesthesia with sevoflurane produces neural toxicity in animals, the precise mechanism of behavioral abnormalities remains elusive [21, 22].

Deposited A*β* protein represents an important neuropathological hallmark of AD pathogenesis [23]. Several studies have shown that sevoflurane exposure of animals significantly increases neuronal apoptosis in several brain regions [21, 24]. Our data displayed that the A*β*_1–42_ level in rat hippocampus significantly increased on day 7 after inhaling sevoflurane, suggesting that inhalation of the anesthetic agent induced neuron loss based on the reports in which the AD-associated A*β* peptide has been shown to induce apoptotic neuronal death [25, 26]. Both the A*β*_1–40_ and A*β*_1–42_ peptides are ubiquitous in biological fluids [27], but A*β*_1–42_ is generally considered to be more pathogenic in AD patients [28, 29]. Given our data, it is reasonable to consider that sevoflurane inhalation caused cognitive decline partly due to intracellular A*β*_1–42_ accumulation that in turn disrupted hippocampal function.

It has been known that GFAP and IBA1 are used as markers of astrocytic and microglial activation [30]. In immunohistochemical studies, GFAP and IBA1 protein expressions in the A*β*-treated hippocampus of rats obviously increased on day 7 after inhaling sevoflurane but not O_2_, suggesting that such exposure mainly acted on these glial cells and aggravated neuroglial activation in the hippocampus. GFAP is an intermediate filament protein, and its immunostaining is largely used to colocalize astrocytes with A*β* plaques [31]. IBA1 as a calcium-binding protein plays an important role in showing microglial activation that is closely associated with nearly all compact deposits of the A*β*-protein found in the senile plaques of AD [32]. Since astrocytes and microglia with the expression of GFAP and IBA1 proteins are distributed in all CA1 layers of the hippocampus [33], it is likely that impaired interplay among these neural cells is responsible for derangements from inhalation anesthesia with sevoflurane to abnormal cognitive processes.

In Western blotting analysis, Bcl-xL, caspase-9, RAGE, and BDNF protein expressions in the A*β*-treated hippocampus tissues were different from the rats exposed to O_2_ and sevoflurane. Caspase-9 and RAGE protein expressions in the A*β*-treated tissues significantly increased, whereas Bcl-xL and BDNF expressions decreased in the rats exposed to sevoflurane as compared to those with O_2_ exposure. These findings led us to conclude that inhalation anesthesia with sevoflurane initiated diverse effects of neural cells in the hippocampus of rats. The pathology of AD is characterized by an accumulation of misfolded proteins and inflammatory changes, which results in region-specific loss of synaptic contacts and neuronal cell death [34]. It has been known that defective control of apoptosis appears to play a central role in the pathogenesis of neurodegenerative diseases [35]. Bcl-xL protects against caspase-9-dependent apoptosis in the nervous system [36]. Targeted disruption of Bcl-xL causes massive death of immature neurons whereas disruption of caspase-9 leads to decreased neuronal apoptosis and neurodevelopmental abnormalities [37]. Since activation of apoptotic death pathways is related to the Bcl-xL-caspase-9 interaction [37], it is reasonable to speculate that sevoflurane exposure induced cognitive deficits and decline probably through potentially disrupting the balance between the Bcl-xL and caspase-9 expression levels in apoptotic cell death regulation.

Inhalation anesthesia with sevoflurane increased RAGE expression and decreased a BDNF level in the A*β*-treated hippocampus, suggesting that the changes in these two protein expressions were partly related to cognitive impairment. RAGE, the receptor of advanced glycation end products, is thought to be one of the potential contributors to the neurodegeneration and neuroinflammation [38, 39]. An increased expression of RAGE is observed in regions of the brain affected by AD [40]. BDNF is one of the neurotrophic factors that play key roles in modulating synaptic transmission, neuronal survival, and axonal guidance, as well as memory formation and cognition [41, 42]. According to experimental data, BDNF protects neurons against A*β*-induced neurotoxicity and contributes to increased A*β* degradation [43]. Since RAGE and BDNF expressions were significantly changed in the animal model, it is very likely that sevoflurane inhalation caused cognitive decline in the processes of learning and memory through triggering neuroinflammation and neurotoxicity in the hippocampus tissue.

Inhalation anesthesia with sevoflurane resulted in increased Il-1*β*, NF-*κ*B, and iNOS mRNA expression in the A*β*-injected hippocampus of rats on day 7, indicating the neuronal inflammatory reactions involved in the hippocampus tissues. IL-1*β* has been considered to be a major proinflammatory cytokine in the onset of inflammatory process and plays a key role in neuronal damage and losses observed in AD [44]. NF-*κ*B has long been considered a prototypical proinflammatory signaling pathway, largely based on the activation by proinflammatory cytokines such as IL-1*β* [45]. In AD patients, iNOS mRNA was increased, suggesting high-output NO production [46], which was consistent with our results. Since blocking or neutralizing IL-1*β* in an AD mouse model largely protects from cognitive deficits [47], it led us to consider that the increased IL-1*β* level would drive potent neuroinflammatory changes contributing to neuronal death in the brain.

Studies have shown that sevoflurane has no damage to normal rats, and in our study similar results were obtained in the rats with injection of NS into the hippocampus. Studies have shown that sevoflurane can exert damage for the aged or neonatal rats, and the results were similar to ours in the A*β*-treated rats, further demonstrating that sevoflurane has an effect on the fragile brain, in which sevoflurane can affect the vulnerable brains.

In conclusion, this study indicates that inhalation anesthesia with sevoflurane causes cognitive deficits and decline through the underlying mechanisms by which neurotoxicity, neuroinflammation, and neuronal apoptosis are developed in the A*β*-injected rat hippocampus.

## Data Availability

The data used to support the findings of this study are available from the corresponding author upon request.
